# Sequential adjustments of task-pair control in dual-task contexts: Examining the role of repetition priming effects at the level of task-pair sets and abstract control states

**DOI:** 10.3758/s13421-025-01694-0

**Published:** 2025-02-18

**Authors:** Patricia Hirsch, Iring Koch, Tilo Strobach

**Affiliations:** 1 Cognitive and Experimental Psychology, Institute of Psychology, RWTH Aachen University, Jägerstr. 17-19, 52066 Aachen, Germany; 2 Department of Psychology, MSH Medical School Hamburg, Am Kasierkai 1, 20457 Hamburg, Germany

**Keywords:** Dual tasks, Dual-task coordination, Task-pair set control, Sequential adjustment

## Abstract

In dual-task situations, two stimuli are presented in rapid succession, requiring participants to perform two tasks simultaneously. Prior studies suggested that when two tasks are performed simultaneously, information about the identity of the two tasks is represented in a joint cognitive representation, referred to as the task-pair set. This evidence comes from studies showing that switching between different task pairs results in performance costs, called task-pair switch costs (i.e., performance in task-pair switches vs. repetitions). In the present study, we focused on the adjustive characteristics of task-pair switching by investigating whether task-pair switch costs are sequentially modulated by the previous experience with a task-pair switch (vs. repetition). First, we reanalyzed the data of four published experiments and observed a reduction of task-pair switch costs after a task-pair switch trial relative to after a task-pair repetition trial. Second, we confirmed this novel finding in a new experiment. The new experiment also showed that performance in a current task-pair repetition was better after a task-pair repetition than after a task-pair switch, whereas the performance in a current task-pair switch was not modulated by the task-pair sequence in the previous trial. These findings suggest that automatic bottom-up repetition priming at the level of task-pair sets, rather than repetition priming at the level of abstract control states, contributes to the sequential adjustment of task-pair switch costs.

## Introduction

When dealing with two tasks at the same time, performance in one or both of these tasks is typically impaired. This is indicated by increased reaction times (RTs) and/or error rates relative to tasks that are executed in isolation (i.e., single tasks; e.g., Jentzsch et al., 2007; Schubert et al., 2008; see, e.g., Fischer & Janczyk, 2022, for a review). A major goal of dual-task research is to understand the cognitive basis of these dual-task costs, which in turn can help to optimize performance (e.g., Fischer & Plessow, 2015). To improve our understanding of the cognitive mechanisms underlying dual-task performance, the present study focuses on higher-level task-coordination processes and examines whether these processes can be flexibly adjusted to changing dual-task demands. The investigation of flexible adjustments of higher-level task-coordination processes in dual tasks can also inform cognitive control theories regarding how cognitive control in complex task environments is regulated in a dynamic, compensatory, and time-varying manner (e.g., Braem et al., 2019).

### The PRP paradigm and theoretical accounts of the PRP effect

A well-controlled procedure for investigating performance limitations in dual-task situations is the psychological refractory period (PRP) paradigm (see, e.g., Pashler, 1994, for a review). In this paradigm, two stimuli are presented in rapid succession. The stimuli are associated with separate RT tasks—component Task 1 (T1) and component Task 2 (T2)—and separate responses on these tasks. The main manipulation relates to the stimulus-onset asynchrony (SOA), which is the time interval between the onsets of the stimulus for T1 and the stimulus for T2. Usually, RTs in T2 (RT2) increase with decreasing SOA. Thus, RT2 is higher with increased temporal overlap in T1 and T2 processing than with no or less temporal overlap (see Strobach et al., 2015, for SOA effects in RT1). This finding is referred to as the PRP effect.

Traditionally, the PRP effect has been attributed to a capacity-limited processing bottleneck at the stage of response selection (see Koch et al., 2018, for a review). According to one of the most prominent models, the bottleneck is structural in nature and allows response selection for only one task at a time (see Pashler, 1994, for a review). When T1 and T2 processing temporally overlaps, response selection for T2 is postponed until the T1 response is selected, resulting in an increase in RT2. In contrast, other theoretical accounts assume that the bottleneck reflects a strategic decision to use serial processing in order to optimize dual-task performance (e.g., Logan & Gordon, 2001; Meyer & Kieras, 1997).

The response-selection bottleneck idea resulted in a venerable research tradition in which numerous studies have been conducted to characterize the nature and source of the processing bottleneck (e.g., Hirsch et al., 2018a; Koch & Prinz, 2002; see e.g., Fischer & Janczyk, 2022; Koch et al., 2018, for reviews). In this research tradition, higher-level cognitive processes beyond the response-selection bottleneck, such as dual-task coordination processes, have, however, received little attention so far.

### Task coordination in dual-task contexts

Dual-task situations require some kind of cognitive mechanisms to coordinate the processing streams of the two component tasks and to regulate their relation to each other (e.g., Strobach et al., 2012). More precisely, the cognitive processes involved in stimulus perception, response selection, and response execution for T1 have to be coordinated with those for T2 (e.g., Lussier et al., 2012; see also Logan & Gordon, 2001, for a computational model incorporating such coordination processes). To coordinate the processing streams for both component tasks, participants need, for instance, information about which two component tasks they have to perform in a given dual-task situation.

There is evidence that dual-task processing relies on a cognitive representation that contains information about the identity of the two component tasks. This evidence comes from PRP studies using the task-pair switching logic (e.g., Hirsch et al., 2017, 2021; see Hirsch & Koch, 2024, for a review). In this logic, three tasks (A, B, & C) are combined in two task-pairs (i.e., PRP trials). T1 is constant and T2 varies across task pairs (e.g., task-pair 1 with Task C as T1 and Task A as T2 [CA]; task-pair 2 with Task C as T1 and Task B as T2 [CB]). The task-pair sequence is varied on a trial-by-trial basis within experimental blocks, and a cue at the beginning of each trial indicates the relevant task-pair.

With this logic, performance in T1 and T2 is typically impaired in task-pair switch trials (e.g., task-pair 1 ➔ task-pair 2) compared with task-pair repetition trials (e.g., task-pair 2 ➔ task-pair 2; e.g., Hirsch et al., 2017), resulting in *task-pair switch costs*. Similar findings were produced in a reversed situation, when T1 varies across task pairs and T2 is constant (e.g., Hirsch et al., 2018b). Note that task-pair switch costs are not attributable to switching between T2 and T1 across trials because there is a task switch between T2 in trial *n* − 1 and T1 in trial *n* in both task-pair switch trials (e.g., CA in trial *n* − 1 and CB in trial *n*) and task-pair repetition trials (e.g., CB in trial *n* − 1 and CB in trial *n*; see Table 1).

**Table 1:** Transitions at the level of task-pairs and component tasks in the task-pair switching logic

Task-pair sequence	Transition at the task-pair level	Transition of tasks across task-pairs (T2 in *n* − 1 and T1 in *n*)
Varying T1 and constant T2		
AC ➔ AC	Repetition	Switch
BC ➔ BC	Repetition	Switch
BC ➔ AC	Switch	Switch
AC ➔ BC	Switch	Switch
Constant T1 and varying T2		
CA ➔ CA	Repetition	Switch
CB ➔ CB	Repetition	Switch
CA ➔ CB	Switch	Switch
CB ➔ CA	Switch	Switch

According to Hirsch and colleagues (2021), task-pair switch costs indicate that the identities of T1 and T2 are jointly represented in a single mental representation, called the *task-pair set*. The task-pair set is conceptualized as an explicit representation which is organized at a hierarchically higher level than the representations of the information concerning the component tasks. The representations of the component tasks are referred to as T1 and T2 “task sets.” It is assumed that the T1 and T2 task sets include task-specific information, such as information on the stimulus–response mappings and task goals (see Kiesel et al., 2010; Koch & Kiesel, 2022; Vandierendonck et al., 2018, for a discussion of this concept). Since the T1 and T2 identities have to be known before the T1 and T2 task sets can be selected, the hierarchy between task-pair sets and the task sets of T1 and T2 is defined as a temporal precedence of information concerning the task pair.

Hirsch and colleagues (2021) proposed that the execution of a dual task presupposes the activation of an appropriate task-pair set in working memory and that two or more task-pair sets cannot be activated simultaneously in working memory, at least not at the same activation level. Whereas in task-pair repetition trials, the task-pair set used in the previous trial is still relevant, in task-pair switch trials, the previous task-pair set cannot be employed again. According to Hirsch and colleagues (2021), task-pair switch costs reflect the time needed to implement a new task-pair set in working memory. In addition to this top-down cognitive control process, memory-based after-effects might contribute to task-pair switch costs. In particular, in task-pair switch trials, the persisting activation of the task-pair set used in the previous trial might positively prime the unintended task-pair set, leading to interference between task-pair sets (see also Allport et al., 1994; Allport & Wylie, 1999). Additional interference can arise due to prior inhibition of the previously unintended but now relevant task-pair set (see, e.g., Hirsch et al., 2017, for a discussion). To gain a better understanding of the cognitive mechanisms underlying task-pair switch costs, it is important to examine how flexible adjustments of cognitive control affect these performance costs.

### Adjustments of dual-task coordination processes

To examine adjustments of cognitive control, numerous single-task studies analyzed the sequential modulation of congruency effects in response-conflict tasks, such as the stroop task (Stroop, 1935), flanker task (Eriksen & Eriksen, 1974), and simon task (Simon & Small, 1969). In response-conflict tasks, each stimulus has task-relevant and task-irrelevant dimensions. For example, Stroop stimuli are color words which are presented in different ink colors. The task is to name the ink color and to ignore the meaning of the word stimulus. A stimulus is congruent when the ink color matches the color word (e.g., BLACK in black letters) and incongruent when it does not (e.g., GREEN in black letters). The congruency effect reflects impaired performance for incongruent stimuli compared with congruent stimuli and is often interpreted as a marker of response conflicts (e.g., Steinhauser & Hübner, 2009).

Several studies showed that the congruency effect in a given trial *n* is smaller after an incongruent stimulus than after a congruent stimulus in trial *n* − 1 (e.g., Blais et al., 2014; Schmidt & Houwer, 2011). This sequential modulation of congruency effects, also termed the Gratton effect (Gratton et al., 1992), has been interpreted as reflecting conflict-driven adjustments of cognitive control (i.e., conflict adaptation, see Botvinick et al., 2001; however, see, e.g., Braem et al., 2019; Egner, 2007, for alternative explanations).

Researchers suggested that processing conflicts trigger a temporary upregulation in cognitive control to overcome the conflict (e.g., Botvinick et al., 2001). In response-conflict tasks, the upregulation results in a stronger activation of task-relevant stimulus information and/or stronger inhibition of task-irrelevant stimulus information. This increase in cognitive control persists even after the completion of a task, thereby reducing a response conflict in the following trial. At the same time, congruent stimuli trigger a relaxation of cognitive control. The relaxation increases the impact of task-irrelevant stimulus information, thereby facilitating performance in congruent trials (Berger et al., 2019). As a consequence, the congruency effect is reduced after incongruent trials relative to congruent trials.

Performance patterns similar to those reported after response conflicts in single-task contexts were also found in dual-task settings (e.g., Durst & Janczyk, 2019; Fischer et al., 2010; Mahesan et al., 2021; Olszanowski et al., 2015). For instance, in a PRP study, Janczyk (2016) examined sequential adjustments of the backward crosstalk effect (i.e., BCE). The BCE reflects worse T1 performance in incompatible trials, where the features of the T1 response do not overlap with those of the T2 response (e.g., left key press in T1 and vocal response “right” in T2), than in compatible trials, where the response features overlap for both tasks (e.g., left key press in T1 and vocal response “left” in T2; see also Hommel, 1998; Lien et al., 2007; Röttger & Haider, 2017). This effect suggests that the response features of T2 are already activated during T1 response selection, leading to a response conflict in incompatible trials. Janczyk (2016) observed that the BCE was reduced (i.e., absent or reversed) after incompatible trials relative to compatible trials, indicating that task shielding against influences of T2 during T1 response selection was adjusted after a response conflict in the previous trial.

Studies on dual-task coordination also reported sequential adjustments of dual-task performance. These studies focused on task-order control in dual-task processing and analyzed *order-switch costs*. Order-switch costs refer to the finding that when participants perform PRP trials with different orders of the component tasks (e.g., order 1 with Task A as T1 and Task B as T2 [AB]; order 2 with BA), T1 and T2 performance is usually worse in order-switch trials (e.g., order 1 ➔ order 2) than in order-repetition trials (e.g., order 2 ➔ order 2). These costs suggest that information about the order in which the component tasks have to be executed is cognitively represented as an order set (e.g., Kübler et al., 2018, 2022a, b; Luria & Meiran, 2003, 2006; Strobach et al., 2021b; Szameitat et al., 2006; see also Schubert, 2008). Similar to the control of task-pair sets, it is assumed that order-set control relies on top-down cognitive processes.

Recently, it has been shown that order-switch costs were smaller after an order switch than after an order repetition (e.g., Steinhauser et al., 2024; Strobach, 2024b; Strobach et al., 2021a, 2023; Strobach & Wendt, 2022; for an overview, see Strobach, 2024a). To investigate the cognitive mechanisms underlying this effect, Strobach and Wendt (2022) varied the intertrial interval (ITI). They found no effect of the ITI on the sequential adjustment of order-switch costs. This temporal persistence is not in line with a short-lived, decaying order set. Rather, it suggests that top-down preparation for the upcoming order of component tasks is robust over some time interval and contributes to the sequential adjustment of order switch costs. Therefore, the authors concluded that the detection of a task-order mismatch (e.g., an order switch trial followed by an order repetition trial) triggers control processes that prepare the cognitive system for an additional switch.

In sum, there is evidence that dual-task coordination processes are affected by previous control demands. So far, PRP studies have shown this effect for BCE control and task-order control. Note, however, that task-order control has to be distinguished from task-pair set control. Both approaches are suitable for examining task coordination in dual tasks, but the approaches focus on different aspects of dual-task coordination.

When using the order-switching paradigm, the emphasis is on the examination of task-order control. To specify the order of the component tasks, one has to know the identities of T1 and T2. Thus, information about task identity is implicitly included in order sets, and task-pair coordination is probably part of order-switch costs. The specific task-pair switch cost, however, cannot be isolated with the order-switching paradigm because in the order-switching paradigm, participants perform the same pair of tasks with a varying order across trials (e.g., AB ➔ BA vs. BA ➔ BA).

In contrast, with the task-pair switching logic, the emphasis is on task-specific information in terms of the T1 and T2 identities. Knowledge of the T1 and T2 identities provides information about the order of the tasks. Hence, task-pair switch costs comprise implicit information on the task order, and order-switch costs are probably part of task-pair switch costs. However, with the task-pair switching logic, pure order-switch costs cannot be isolated because the order of one component task is held constant across trials, whereas the identity of the other task is manipulated across trials (e.g., CA ➔ CA vs. CB ➔ CA).

Thus, the order-switching paradigm and the task-pair switching logic have complementary strengths and can be used to examine different research questions on dual-task coordination, namely questions related to task-order set control and questions related to task-pair set control, respectively.^1^ Sequential adjustments of order-switch costs suggest that similar control processes may also work at the level of task-pair sets. However, it is unclear whether task-pair switch costs show sequential adjustment effects.

### The present study

The aim of this study was to examine whether task-pair set control, as a specific type of dual-task coordination, is susceptible to sequential flexible adjustments. To this end, we first analyzed task-pair switch costs in trial *n* as a function of the task-pair sequence in trial *n* − 1, using the data of four published task-pair switching experiments (i.e., Experiments 1 to 4; Hirsch et al., 2018b; Hirsch et al., 2021). We hypothesized that in contrast to task-pair repetition trials, in task-pair switch trials, the irrelevant task-pair is more strongly activated than the relevant task-pair, leading to a conflict at the level of selecting task-pair sets. Moreover, we predicted that the task-pair selection conflict in task-pair switch trials initiates a regulation of (task-pair) cognitive control, allowing the cognitive system to resolve future conflicts more efficiently, as indicated by reduced task-pair switch costs in the following trial. Hence, in contrast to typical conflict adaptation studies that focus on response conflicts (i.e., measured as congruency effects), the present study examined the effect of conflicts at the level of selecting cognitive task representations.

As the published experiments for the data reanalysis differed in various aspects, we also aimed to explore which factors influence the sequential adjustment of task-pair switch costs. For this purpose, we included *experiment* as a between-subjects variable in our design. Second, we conducted a new experiment (i.e., Experiment 5) to replicate the findings of the data reanalysis and to examine in more detail the cognitive mechanisms underlying the sequential adjustment of task-pair switch costs.

## Experiments 1–4: Data reanalysis of published experiments

The data reanalysis only included studies with two task-pairs that showed robust task-pair switch costs and had at least 20 trials per cell (see also Simmons et al., 2011) for an additional post hoc analysis of potential adjustments of task-pair switch costs by the previous task-pair sequence. Taking these criteria into account, the data collected in the studies by Hirsch et al. (2018b; Experiment 1 & Experiment 2) and Hirsch et al. (2021, Experiment 1 & Experiment 2) provided an adequate basis for analyzing the adjustment of task-pair switch costs in trial *n* by the task-pair sequence in trial *n* − 1. In the following, we describe the general methodological characteristics of these experiments, followed by experiment-specific variations.

### General method

#### Participants

In each experiment, the final sample size comprised 24 participants (Experiment 1: 23 women, 18 right-handed, *M* = 22.0 years, *SD* = 3.2; Experiment 2: 20 women, 21 right-handed, *M* = 23.5 years, *SD* = 2.4; Experiment 3: 20 women, 22 right-handed, *M* = 20.6 years, *SD* = 2.7; Experiment 4: 17 women, 21 right-handed, *M* = 22.7 years, *SD* = 3.7). According to self-reports, they had normal or corrected-to-normal vision and no hearing impairments. All participants received partial course credit for their participation.

#### Stimuli, tasks, and responses

The stimulus material contained visual task-pair cues, a fixation cross, eight pictures, and two tones. The cues and the fixation cross were displayed in 28-point, black Arial font. The pictures had a size of 7 cm × 6 cm and showed either a black can or a black cup in upright or upside-down orientation with a handle on the left or right side (see Hirsch et al., 2017, for the pictures of the cans and cups). All visual stimuli were presented in the center of a white 17-in. screen. The tones, a 200-Hz and a 600-Hz tone, were presented via headphones.

The tones were associated with a tone categorization task. Responses were made with two horizontally arranged response keys of a QWERTZ keyboard. Low-pitch tones required a response with the left key and high-pitch tones with the right key. Responses were entered with the middle and index fingers of one hand. Due to the spatial–musical association of response codes effect (e.g., Keller & Koch, 2006; Rusconi et al., 2006), the response keys for the tone task were not counterbalanced across participants.

The pictures were associated with three tasks, including a side categorization task, an orientation categorization task, and an object categorization task. In the side task, participants decided whether the handle was on the left or right side of the object, and in the orientation task, whether the object was presented upright or upside down. In the object task, participants categorized the pictures as can or cup. The responses were entered with the index and middle fingers of the other hand (i.e., the hand that was not used for the tone task), using an additional set of two horizontally arranged keys. The keys were counterbalanced across participants for the orientation task and the object task. Taking into account the spatial stimulus–response compatibility effect (i.e., worse performance for spatially incompatible than for spatially compatible stimulus–response mappings; e.g., Fitts & Deininger, 1954), there was no counterbalancing for the side task; participants responded to a left handle with the left key and to a right handle with the right key.

The tone task and the visual tasks were combined in different task-pairs. T1 was constant and T2 varied across task-pairs or vice versa. In the case of a constant T1, there were the following task-pairs: Task-pair 1 with the tone task as T1 and the side task as T2, task-pair 2 with the tone task as T1 and the orientation task as T2, and task-pair 3 with the tone task as T1 and the object task as T2. In the case of a varying T1 and a constant T2, the visual tasks served as T1 and the tone task as T2. In all experiments, participants performed two of the three different task-pairs. T1 required responses with the left hand using the *Y-*key and the *X-*key which were located at the left side of the keyboard. Responses for T2 were made with the right hand using the *N*-key and *M*-key which were located at the right side.

#### Procedure

**Fig. 1:**
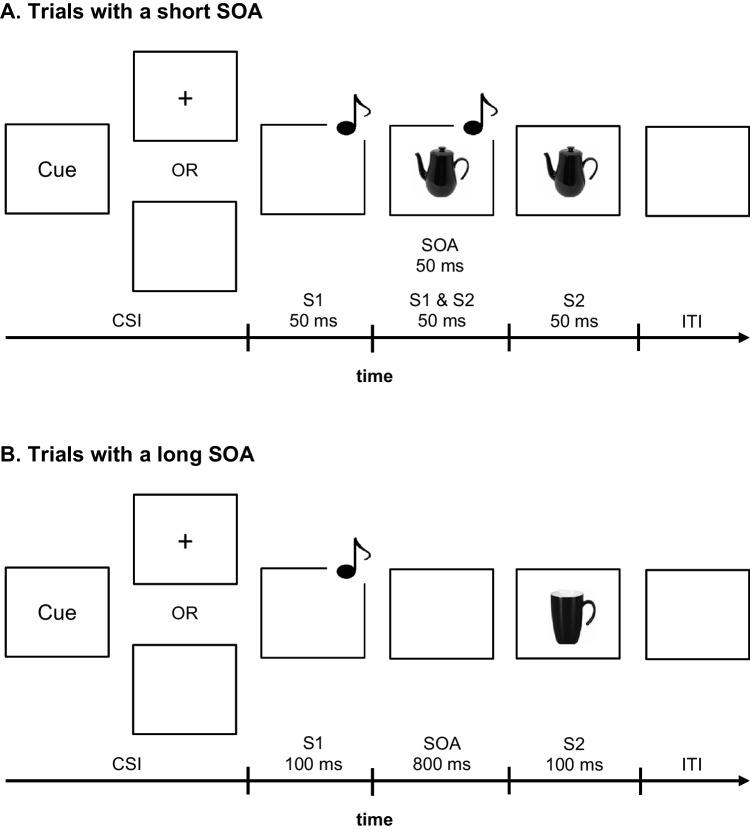
General trial procedure in task-pair switching experiments with a constant Task 1 (T1) and a varying Task 2 (T2) (Experiment 2–5) for **A.** trials with a short stimulus-onset-asynchrony (SOA) of 50 ms and **B.** trials with a long SOA of 800 ms. *Note.* In task-pair switching experiments with a varying T1 and a constant T2, the visual stimulus is presented for T1 and the auditory stimulus for T2 (Experiment 1)

The experiments were run in a single session with one participant at a time. At the beginning of the experiments, the instructions were presented on the screen and emphasized speed and accuracy for both tasks. Each trial started with the presentation of a task-pair cue (see Fig. 1). The cue was replaced by a fixation cross in some experiments, whereas other experiments used a blank screen after the cue. After this cue–stimulus interval (CSI), S1 appeared for 100 ms. S2 was presented for 100 ms after a SOA. The task-pair sequence was random with some restrictions (see section on experiment-specific methodological variations).

## Experiment-specific methodological variations

The experiments differed with respect to several design details (for an overview, see Table 2).

**Table 2:** Differences in the sample size (*n*), task-pair composition, cue–stimulus interval (CSI), stimulus-onset asynchrony (SOA), intertrial interval (ITI), cues per task-pair mapping, and go/no-go manipulations across Experiment 1, 2, 3, 4, and 5

	*n*	Task-pair composition	CSI (in ms)	SOA (in ms)	ITI (in ms)	Cues per task-pair	Go/no-go manipulation
**Experiment **1 (Hirsch et al., 2018b)	24	Varying T1 & constant T2	500	50 vs. 800	1,000	1	No
**Experiment **2 (Hirsch et al., 2018b)	24	Constant T1 & varying T2	500	50 vs. 800	1,000	1	No
**Experiment **3 (Hirsch et al., 2021)	24	Constant T1 & varying T2	600	50 vs. 800	1,000	2	No
**Experiment **4 (Hirsch et al., 2021)	24	Constant T1 & varying T2	350 vs. 1,250	50	600 vs. 1,500	1	Yes
**Experiment **5 (present study)	40	Constant T1 & varying T2	500	800	1,000	1	No

### Experiment 1 (Hirsch et al., 2018b, Experiment 1)

The task-pair cues were the German words for *side* (“Seite”), *orientation* (“Orientierung”), and *object* (“Objekt”). T1 was one of the visual tasks and varied across task-pairs. T2 was the tone task and was constant across task pairs. Each participant performed two of the three task-pairs. Which two of the three task-pairs were performed was counterbalanced across participants. There was a CSI of 500 ms (400-ms task-pair cue, followed by a fixation cross for 100 ms). The SOA was 50 ms or 800 ms and varied randomly across trials. Trials were separated by an ITI of 1,000 ms. Participants performed a practice block of 12 trials and five experimental blocks, each including 65 trials. The task-pair sequence was presented randomly with the stipulation that there was the same number of each combination of the task-pair sequence and SOA in each cell of the design (i.e., 80 experimental trials per cell for each participant).

### Experiment 2 (Hirsch, et al., 2018b, Experiment 2)

Experiment 2 was identical to Experiment 1, except that the tone task was the constant T1 and the visual tasks were the varying T2.

### Experiment 3 (Hirsch et al., 2021, Experiment 1)

In contrast to Experiment 1 and Experiment 2, which used one cue per task-pair, in Experiment 3, four cues were combined to two cue pairings in order to dissociate cue repetitions (i.e., identical cue for same task-pair) from task-pair repetitions (i.e., different cues for the same task-pair; see Altmann, 2006, for the same logic in a task-switching study). The first pairing comprised the letters *H* and *U* and the second pairing the letters *L* and *W*. Whereas *H* and *U* introduced task-pair 1, which comprised the tone task as T1 and the side task as T2, *L* and *W* were the cues for task-pair 2, which included the tone task as T1 and the object task as T2. Which cue pairing was used for which task-pair was counterbalanced across participants. Note, however, that in each trial only one cue of the pairing was presented. There was a CSI of 600 ms (i.e., no use of a fixation cross or blank screen) and a random SOA of 50 ms and 800 ms (trial-to-trial manipulation). The ITI was 1,000 ms. Participants performed a practice block of 18 trials, followed by six experimental blocks of 49 trials each. The task-pair sequence was random, resulting in 33.33% task-pair switch trials with a cue switch, 32.99% task-pair repetition trials with a cue switch, and 33.68% task-pair repetition trials with a cue repetition (i.e., mean experimental trials per cell across participants: 48 task-pair switch trials with a cue switch and a short SOA and 48 for a long SOA, 47 task-pair repetition trials with a cue repetition with a short SOA and 48 for a long SOA, 49 task-pair repetition trials with a cue repetition with a short SOA and 48 for a long SOA).

### Experiment 4 (Hirsch et al., 2021, Experiment 2)

The task-pair cues were the German words for side and object. There were two task-pairs with a constant tone task as T1. T2 was either the side task or the object task. The task-pair cues were presented for 250 ms, followed by a blank screen for 100 ms or 1,000 ms. Thus, the CSI was either 350 ms or 1,250 ms and varied randomly from trial to trial. To hold the time interval between the response for T2 and the onset of S1 in the next trial constant across the conditions with a short and long CSI, the ITI was 600 ms or 1,500 ms and was manipulated inversely to the CSI. The SOA was constant at 50 ms. There were go trials and no-go trials. In go-trials, S1 and S2 appeared after the CSI, whereas in no-go trials, the cue was not followed by the presentation of S1 and S2. Hence, participants executed neither the T1 response nor the T2 response during the later trials. There were two practice blocks of 16 trials each. One practice block included go trials and the other comprised both go and no-go trials. After the practice blocks, participants performed nine blocks of 65 trials each; 75.00% of the trials were go-trials and 25.00% no-go trials. The task-pair sequence was presented randomly with the restriction that in experimental trials, there was the same number of each combination of the task-pair sequence and CSI (i.e., 108 experimental go-trials for each cell per participant).

#### Design

We analyzed T1 and T2 performance based on a 2 × 2 × 4 mixed design. The independent within-subjects variables were task-pair sequence in trial *n* (task-pair switch vs. task-pair repetition in trial *n*) and, constituting the novel variable in this reanalysis, task-pair sequence in trial *n* − 1 (task-pair switch vs. task-pair repetition in trial *n* − 1). We also included experiment as a between-subjects variable (Experiments 1, 2, 3, & 4) to examine whether the novel interaction effect between task-pair sequence in trial *n* and task-pair sequence in trial *n* − 1 is affected by experiment-specific variables (i.e., three-way interaction). The theoretically less interesting main effect of experiment and the two-way interactions with experiment (i.e., task-pair sequence in trial *n* and experiment & task-pair sequence in trial *n* − 1 and experiment) are reported in Appendix 1. Generally, we averaged across all other conditions, such as CSI, SOA, ITI, task-pair cue mappings, and go/no-go manipulations in the preceding trial. The dependent variables were RTs and error rates.

### Results

Raw data for all experiments is publicly available on PsychArchives (Experiments 1 & 2: 10.23668/psycharchives.13509, Experiment 3 & 4: http://doi.org/10.23668/psycharchives.3140). For the data analyses, we excluded practice trials, the two first trials in each experimental block, and trials which were not preceded by two correct trials from all analyses. In addition, for the RT analysis, we discarded trials with an error in T1 or T2 and all trials with RTs exceeding 3 standard deviations from a given participant’s mean (Experiment 1: 1.31% in T1 & 0.91% in T2; Experiment 2: 1.04% in T1 & 0.69% in T2; Experiment 3: 1.07% in T1 & 0.83% in T2; Experiment 4: 0.65% in T1 & 0.72% in T2). For the accuracy analysis, we excluded trials with an error in T2 when analyzing T1 and trials with an error in T1 when analyzing T2.

**Fig. 2:**
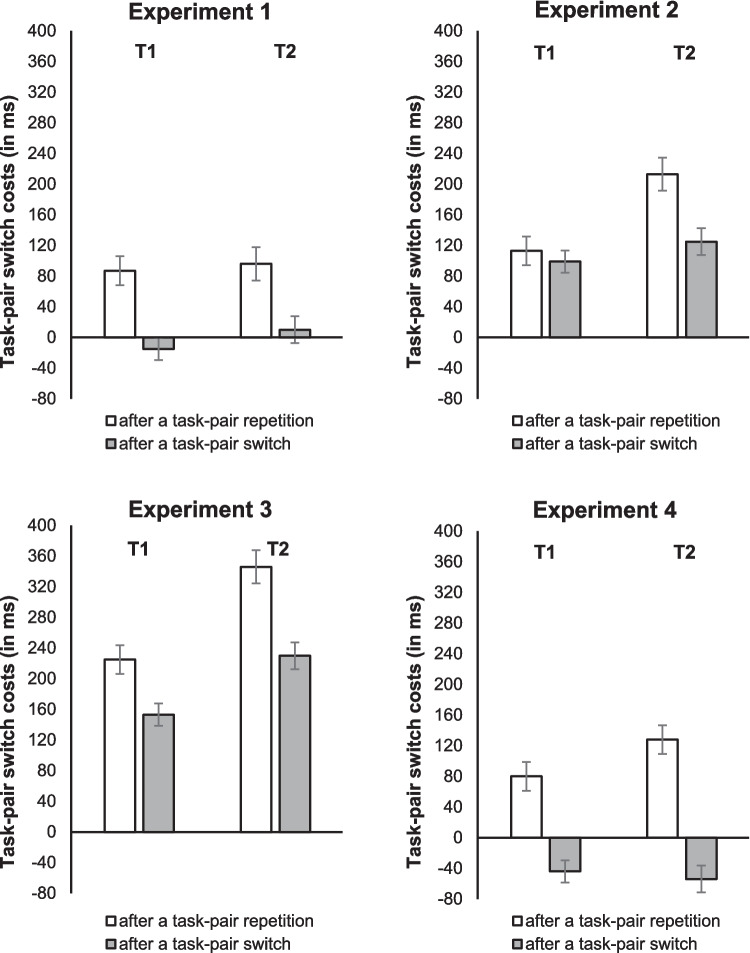
Task-pair switch costs (in ms) in Experiment 1, 2, 3, and 4 for Task 1 (T1; i.e., RT1) and Task 2 (T2; i.e., RT2) as a function of the task-pair sequence in trial *n* − 1 (task-pair switch vs. task-pair repetition in trial* n* − 1). Error bars represent the standard error of the mean

Separate analyses of variance (ANOVAs) were run on mean RTs and error rates in T1 and T2 (see Tables 3 and 4). For between-experiment effects, we additionally conducted post hoc Dunnett-T3 tests. For all other interactions, we used planned simple main effect analyses. Note that due to the violation of the assumption of homogeneity of covariance (all Box’s tests with *p* < .001) and the assumption of equality of error variances (at least one of the Levene’s tests for each ANOVA with *p* < .05), the reliability of the results of the between-experiment comparison may be limited. Figure 2 illustrates the relevant task-pair switch costs of Experiments 1, 2, 3, and 4.

**Table 3:** Reaction times (in ms; standard errors in parenthesis) for Task 1 (T1) and Task 2 (T2) in Experiments 1, 2, 3, and 4 as a function of the task-pair sequence in trial *n *(task-pair switch vs. task-pair repetition in trial *n*) and the task-pair sequence in trial *n* − 1 (task-pair switch vs. task-pair repetition in trial *n* − 1)

	T1		T2	
	Task-pair sequence in trial *n* − 1			
	Task-pair repetition	Task-pair switch	Task-pair repetition	Task-pair switch
**Experiment **1				
Task-pair switch in trial *n*	723 (113.6)	691 (112.6)	964 (118.4)	941 (109.4)
Task-pair repetition in trial *n*	636 (94.4)	706 (96.6)	868 (97.7)	931 (100.1)
Task-pair switch costs	87 (37.6)	−15 (23.8)	96 (43.4)	10 (35.1)
**Experiment **2				
Task-pair switch in trial *n*	1,183 (113.1)	1,208 (105)	1,487 (118.3)	1,506 (97.7)
Task-pair repetition in trial *n*	1,070 (94.4)	1,109 (96.5)	1,274 (97.7)	1,381 (100.8)
Task-pair switch costs	113 (37.1)	99 (29.1)	213 (43.3)	125 (35.0)
**Experiment **3				
Task-pair switch in trial *n*	1,532 (112.4)	1,506 (104.0)	1,941 (120.0)	1,911 (112.0)
Task-pair repetition in trial *n*	1,307 (92.9)	1,353 (95.4)	1,595 (100.3)	1,681 (104.0)
Task-pair switch costs	225 (37.6)	153 (29.1)	346 (43.4)	230 (35.1)
**Experiment **4				
Task-pair switch in trial *n*	938 (113.2)	792 (104.7)	1,166 (118.4)	969 (109.4)
Task-pair repetition in trial *n*	858 (94.4)	836 (97.0)	1,038 (97.6)	1023 (100.1)
Task-pair switch costs	80 (38.0)	−44 (29.0)	128 (37.6)	−54 (34.9)

**Table 4:** Error rates (in percentage; standard errors in parenthesis) for Task 1 (T1) and Task 2 (T2) in Experiments 1, 2, 3, and 4 as a function of the task-pair sequence in trial *n* (task-pair switch vs. task-pair repetition in trial *n*) and the task-pair sequence in trial *n* − 1 (task-pair switch vs. task-pair repetition in trial* n* − 1)

	T1		T2	
	Task-pair sequence in trial *n* − 1			
	Task-pair repetition	Task-pair switch	Task-pair repetition	Task-pair switch
**Experiment **1				
Task-pair switch in trial *n*	6.65 (0.86)	6.80 (0.75)	5.11 (1.15)	6.94 (1.20)
Task-pair repetition in trial *n*	4.14 (0.66)	5.92 (0.74)	6.03 (0.99)	5.71 (1.02)
Task-pair switch costs	2.51 (0.74)	0.88 (0.80)	−0.92 (1.12)	1.23 (1.00)
**Experiment **2				
Task-pair switch in trial *n*	2.99 (0.86)	3.49 (0.75)	9.80 (1.15)	11.36 (1.20)
Task-pair repetition in trial *n*	3.01 (0.66)	4.24 (0.74)	6.64 (0.99)	8.99 (1.02)
Task-pair switch costs	−0.02 (0.74)	−0.75 (0.80)	3.16 (1.12)	2.37 (1.00)
**Experiment **3				
Task-pair switch in trial *n*	1.58 (0.86)	2.23 (0.75)	9.54 (1.15)	8.38 (1.20)
Task-pair repetition in trial *n*	2.90 (0.66)	2.06 (0.74)	5.87 (0.99)	7.79 (1.02)
Task-pair switch costs	−1.32 (0.74)	0.17 (0.80)	3.67 (1.12)	0.59 (1.00)
**Experiment **4				
Task-pair switch in trial *n*	4.10 (0.86)	2.99 (0.75)	9.86 (1.15)	7.84 (1.20)
Task-pair repetition in trial *n*	3.79 (0.66)	3.87 (0.74)	5.34 (0.99)	7.71 (1.02)
Task-pair switch costs	0.31 (0.74)	−0.88 (0.80)	4.52 (1.12)	0.13 (1.00)

#### RT1

The ANOVA showed a main effect of task-pair sequence in trial *n*, *F*(1,92) = 35.38, *p* < .001, η_p_^2^ = .28, indicating that RT1 was longer when the current trial was a task-pair switch trial than when it was a task-pair repetition trial (1,072 ms vs. 985 ms; task-pair switch costs: 87 ms). The main effect of task-pair sequence in trial *n* − 1 was not significant, *F*(1,92) = 0.54, *p* = .466, η_p_^2^ = .01. However, as can be seen in the left “T1” part in each panel of Fig. 2, the interaction of task-pair sequence in trial *n* and task-pair sequence in trial *n* − 1 was significant, indicating that task-pair switch costs in a current trial were reduced after a task-pair switch compared with a task-pair repetition (49 ms vs. 126 ms), *F*(1,92) = 22.31, *p* < .001, η_p_^2^ = .20. A set of planned simple main effects analyses showed that task-pair switch costs in a current trial were significant after both a task-pair switch trial,* F*(1,92) = 11.14, *p* = .001, η_p_^2^ = .11, and a task-pair repetition trial *F*(1,92) = 44.90, *p* < .001, η_p_^2^ = .33. The interaction of task-pair sequence in trial *n*, task-pair sequence in trial *n* − 1, and experiment was nonsignificant, *F*(3,92) = 2.14, *p* = .10, η_p_^2^ = .07.

#### Error rates in T1

For the error rates in T1, neither the main effect of task-pair sequence in trial *n*, *F*(1,92) = 0.12, *p* = .665, η_p_^2^ < .01, nor the main effect of task-pair sequence in trial *n* − 1 was significant,* F*(1,92) = 1.28, *p* = .261, η_p_^2^ = .01 (see Table 4). The interaction of task-pair sequence in trial *n* and task-pair sequence in trial *n* − 1, *F*(1,92) = 0.85, *p* = .359, η_p_^2^ = .01, and the interaction of task-pair sequence in trial *n*, task-pair sequence in trial *n* − 1, and experiment, *F*(3,92) = 1.53, *p* = .211, η_p_^2^ = .05, were nonsignificant, too.

#### RT2

The ANOVA on RT2 yielded a significant main effect of task-pair sequence in trial *n*, *F*(1,92) = 66.46, *p* < .001, η_p_^2^ = .42. RT2 was longer in task-pair switch trials than in task-pair repetition trials (1,360 ms vs. 1,224 ms), resulting in task-pair switch costs of 136 ms. The main effect of task-pair sequence in trial *n* − 1 was nonsignificant, *F*(1,92) = 0.02, *p* = .893, η_p_^2^ < .001, but the interaction of task-pair sequence in trial *n* and task-pair sequence in trial *n* − 1 was significant, *F*(1,92) = 32.68, *p* < .001, η_p_^2^ = .26. As shown in the right “T2” part of each panel of Fig. 2, task-pair switch costs in a current trial were reduced after a task-pair switch compared with a task-pair repetition (78 ms vs. 196 ms). A set of planned simple main effects analyses demonstrated that like for RT1, task-pair switch costs in a current trial were significant after both a task-pair switch, *F*(1,92) = 19.78, *p* < .001, η_p_^2^ = .18, and a task-pair repetition, *F*(1,92) = 81.48, *p* < .001, η_p_^2^ = .47. The interaction of task-pair sequence in trial *n*, task-pair sequence in trial *n* − 1, and experiment was nonsignificant, *F*(3,92) = 1.20, *p* = .315, η_p_^2^ = .04.

#### Error rates for T2

The ANOVA showed a significant main effect of task-pair sequence in trial *n*, *F*(1,92) = 23.27, *p* < .001, η_p_^2^ = .20, with higher error rates in task-pair switch trials than in task pair repetition trials (8.60% vs. 6.76%). The main effect of task-pair sequence in trial *n −* 1 was also significant, *F*(1,92) = 4.00, *p* = .048, η_p_^2^ = .07. Error rates were higher after task-pair switch than after task-pair repetition (8.09% vs. 7.27%). The interaction of task-pair sequence in trial *n* and task-pair sequence in trial *n* − 1 was significant, too, *F*(1,92) = 4.24, *p* = .04, η_p_^2^ = .04. As for RTs, task-pair switch costs in a current trial were reduced after a task-pair switch compared with a task-pair repetition (1.03% vs. 2.61%). A set of planned simple main effects analyses showed that task-pair switch costs in a current trial were significant after both a task-pair switch, *F*(1,92) = 4.638, *p* = .034, η_p_^2^ = .048, and a task-pair repetition, *F*(1,92) = 21.56, *p* < .001, η_p_^2^ = .19.

Moreover, the ANOVA yielded a significant interaction of task-pair sequence in trial *n*, task-pair sequence in trial *n −* 1, and experiment, *F*(3,92) = 3.75, *p* = .014, η_p_^2^ = .11, indicating that the sequential adjustment of task-pair switch costs was significantly larger in Experiment 4 than in Experiment 1 (*p <* .001; all other *p* values > .16). In addition, simple main effect analyses showed that in Experiment 1 and Experiment 2, task-pair switch costs after a task-pair switch did not differ from task-pair switch costs after a task-pair repetition, *F*(1,92) = 2.11, *p* = .15, η_p_^2^ = .02 and *F*(1,92) = 0.28, *p* = .596, η_p_^2^ < .01. However, task-pair switch costs were lower after a task-pair switch than after a task-pair repetition in Experiment 3 (reduction of task-pair switch costs: 3.08%), *F*(1,92) = 4.33, *p* = .04, η_p_^2^ = .05, and Experiment 4 (reduction of task-pair switch costs: 4.39%), *F*(1,92) = 8.76, *p* = .004, η_p_^2^ = .09.

### Discussion

In the data reanalysis of Experiment 1 to 4, we observed task-pair switch costs and, most notably, we also found a sequential adjustment of task-pair switch costs in terms of a reduction of these costs after a task-pair switch compared with after a task-pair repetition. In sum, these findings suggest that task-pair set control can be adjusted to processing demands in the previous trial.

## Experiment 5: New data and tests of specific hypotheses on adjustment mechanisms

In Experiment 5, we aimed to confirm the sequential adjustment of task-pair switch costs by the task-pair sequence in trial *n* − 1 in a new experiment and to examine the cognitive mechanisms underlying the sequential adjustment of these costs more elaborately. We employed Experiment 2 of the data reanalysis as a basis for the method in Experiment 5 because Experiment 2 showed the weakest sequential adjustment of task-pair switch costs in T1 across the experiments, thereby representing a conservative baseline to test the adjustments of these costs.^2^ Applying some conditions (e.g., constant T1) of the former Experiment 2 in the new Experiment 5 is therefore the most diagnostic test of flexible adjustments of task-pair control. Moreover, in the new Experiment 5, we used only the long SOA condition from Experiment 2 because this SOA condition is consistent with conditions investigating task-order control (e.g., Strobach et al., 2021a). Thus, this decision increases the possibilities to draw conclusions from the present task-pair switching logic to the order-switching paradigm.

To get insights into the cognitive mechanisms underlying the sequential adjustment of task-pair switch costs by the task-pair sequence in the previous trial, we tested two accounts. For this purpose, we analyzed whether the performance in (1) a current task-pair repetition and (2) a current task-pair switch differed depending on whether the previous trial contained a task-pair switch or task-pair repetition.

The first account is related to *task-pair transition sets* (see Steinhauser et al., 2024, for a similar idea in order control). A transition set is a cognitive representation of abstract control states. Control states are assumed to reflect control parameters needed to meet the processing demands in a current trial—namely, to switch or to repeat a task-pair. They are abstract because they are independent of the concrete task-pairs they operate on (e.g., Dignath & Kiesel, 2021; Egner, 2023). More specifically, there might be a task-pair switch transition set including the control parameters for a task-pair switch and a task-pair repetition transition set encompassing the control parameters for a task-pair repetition. Performance should be worse in trials in which participants need to implement a new task-pair transition set in working memory compared with trials in which participants can apply the previous task-pair transition set again. Accordingly, any repetition of the task-pair transition set should result in a performance benefit, meaning that the reduction of task-pair switch costs after a task-pair switch relative to a task-pair repetition should be driven by enhanced performance in a task-pair switch after a task-pair switch relative to a task-pair repetition and facilitated performance in a task-pair repetition after a task-pair repetition compared with a task-pair switch. Thus, this account explains sequential adjustments of task-pair switch costs by repetition priming of abstract control states.

Yet, in addition or instead of repetition priming of abstract control states, sequential adjustments of task-pair switch costs might also be attributable to repetition priming effects at the level of task-pair sets. More precisely, a task-pair set might remain activated after the completion of a dual task, and the persisting activation of a task-pair set might result in positive priming of this task-pair set when the task-pair is relevant again (see also Hirsch et al., 2017). Following this account, the sequential adjustment of task-pair switch costs should be mainly driven by facilitated performance in task-pair repetitions after a task-pair repetition compared with after a task-pair switch, whereas performance in a task-pair switch should not differ as a function of the task-pair sequence in the previous trial. Thus, the sequential adjustment of task-pair switch costs should be attributable to a facilitation of sequential task-pair repetitions (see also Meiran et al., 2010, for a similar notion in task switching).

Hence, an account assuming repetition priming of abstract control states predicts a task-pair switch benefit after a task-pair switch and a task-pair repetition benefit after a task-pair repetition. In contrast, an account assuming repetition priming of task-pair sets predicts a task-pair repetition benefit after a task-pair repetition but no effect of the task-pair sequence in the previous trial on the performance in a current task-pair switch trial.

### Method

#### Participants

A new group of 40 participants (29 women; 34 right-handed; *M* = 23.4 years; *SD* = 3.4) with normal or corrected-to-normal vision and no hearing impairments participated in Experiment 5. All participants gave written informed consent before the experiment. The procedures performed in the present experiment were in accordance with the 1964 Helsinki declaration and its later amendments or comparable ethical standards. Since we tested only healthy young adults, and no physical or psychological discomfort and harm due to the participation in this experiment could reasonably be expected, the study did not need to be approved by an ethics committee.

The sample size was estimated in G*Power (Faul et al., 2007) for the interaction effect of task-pair sequence in trial *n* and task-pair sequence in trial *n* − 1 for an ANOVA with repeated factors. We determined the effect size based on the results of the data reanalysis. Since the interaction was more consistent in the RT data than in the error data (i.e., effects for T1 and T2 in RTs vs. effect in the error rates for T2, but no effect for T1), we averaged across the effect sizes observed for RT1 and RT2. The analysis revealed that 40 participants would allow us to detect a medium-sized interaction effect of *f* = 0.27 with a α level of .05 and a statistical power of 0.90.

#### Stimuli, tasks, responses, and procedure

The stimuli, tasks, responses, and procedure were identical to those used in Experiment 2 of the data reanalysis, except that we replaced trials including a short SOA with trials comprising a long SOA of 800 ms. Thus, in Experiment 5, there were only trials with a long SOA. The task-pair sequence varied randomly with the restriction of a balanced number of task-pair switches and task-pair repetitions (i.e., 160 experimental trials per cell for each participant).

#### Design

Performance in T1 and T2 was analyzed based on a 2x2 repeated-measures design with the independent within-subjects variables task-pair sequence in trial *n* (task-pair switch vs. task-pair repetition in trial *n*) and task-pair sequence in trial *n* − 1 (task-pair switch vs. task-pair repetition in trial *n* − 1). The dependent variables were RTs and error rates.

### Results

**Fig. 3:**
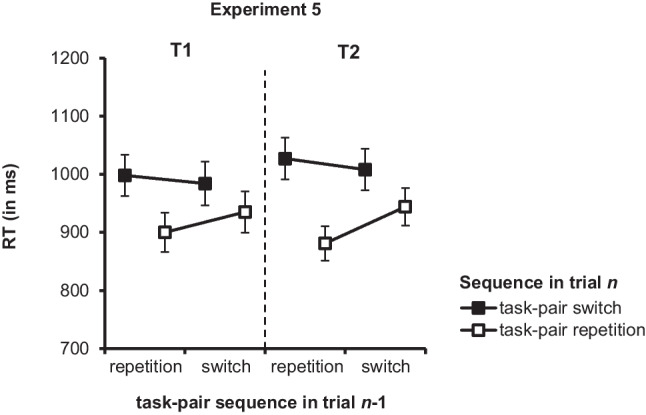
Reaction times (RT in ms) in Experiment 5 for Task 1 (T1; RT1) and Task 2 (T2, RT2) as a function of task-pair sequence in trial *n* (task-pair switch vs. task-pair repetition in trial *n*) and task-pair sequence in trial *n* − 1 (task-pair switch vs. task-pair repetition in trial* n* − 1). Error bars represent the standard error of the mean

Raw data for this experiment is publicly available on PsychArchives (10.23668/psycharchives.15957). Using the same criteria as in the data reanalysis, we excluded 1.16% outliers in T1 and 1.27% in T2 and ran separate ANOVAs on mean RTs and error rates (see Fig. 3 and Table 5). To examine how performance in a current trial was affected by the task-pair sequence in trial *n* − 1, we computed an additional set of planned simple main effect analyses (i.e., performance in a current task-pair switch trial as a function of the task-pair sequence in trial *n* − 1 and performance in a current task-pair repetition trial as a function of the task-pair sequence in trial *n* − 1). The results of these analyses allowed for conclusions about the role of automatic priming effects in the sequential adjustment of task-pair switch costs.^3^

**Table 5:** Mean error rates (in percentage; standard errors in parenthesis) for Task 1 (T1) and Task 2 (T2) in Experiment 5 as a function of the task-pair sequence in trial *n* (task-pair switch vs. task-pair repetition in trial *n*) and the task-pair sequence in trial *n* − 1 (task-pair switch vs. task-pair repetition in trial* n* − 1)

	T1		T2	
	Task-pair sequence in trial *n* − 1			
Experiment 5	Task-pair repetition	Task-pair switch	Task-pair repetition	Task-pair switch
Task-pair switch in trial *n*	4.52 (0.88)	4.10 (0.69)	11.49 (1.17)	10.81 (1.13)
Task-pair repetition in trial *n*	4.03 (0.66)	3.53 (0.59)	5.88 (0.84)	8.48 (0.91)
Task-pair switch costs	0.49 (0.69)	0.57 (0.61)	5.61 (1.06)	2.33 (0.87)

#### RT1

The ANOVA showed a main effect of task-pair sequence in trial *n*, *F*(1,39) = 22.73, *p* < .001, η_p_^2^ = .37. RT1 was longer in task-pair switch trials than in task-pair repetition trials (991 ms vs. 918 ms), reflecting task-pair switch costs of 73 ms (see left “T1” part of Fig. 3). The main effect of task-pair sequence in trial *n* − 1 was not significant, *F*(1,39) = 0.76, *p* = .338, η_p_^2^ = .02, but the interaction of task-pair sequence in trial *n* and task-pair sequence in trial *n* − 1 was significant, indicating that task-pair switch costs in a current trial were reduced after a task-pair switch compared with after a task-pair repetition (49 ms vs. 98 ms), *F*(1,39) = 4.62, *p* = .038, η_p_^2^ = .11.

A first set of planned simple main effects analyses revealed that task-pair switch costs in the current trial were significant after both a task-pair switch (49 ms), *F*(1,39) = 11.11, *p* = .002, η_p_^2^ = .22, and a task-pair repetition (98 ms), *F*(1,39) = 18.37, *p* < .001, η_p_^2^ = .32. More importantly, a second set of planned simple main effect analyses showed that RT1 in a current task-pair repetition trial was shorter when the previous trial was also a task-pair repetition than when it was a task-pair switch (difference: 35 ms), *F*(1,39) = 6.41, *p* = .016, η_p_^2^ = .14. However, RT1 in a current task-pair switch trial did not differ statistically as a function of the task-pair sequence in trial *n* − 1, *F*(1,39) = 0.56, *p* = .458, η_p_^2^ = 01.

#### Error rates for T1

The main effect of task-pair sequence in trial *n*, *F*(1,39) = 1.55, *p* = .221, η_p_^2^ = .04, the main effect of task-pair sequence in trial *n* − 1, *F*(1,39) = 0.51, *p* = .481, η_p_^2^ = .01, and the interaction, *F*(1,39) = 0.01, *p* = .937, η_p_^2^ < .001, were nonsignificant (see Table 5).

#### RT2

As depicted in Fig. 3 (i.e., right “T2” part), for RT2, the ANOVA yielded a significant main effect of task-pair sequence in trial *n*, *F*(1,39) = 17.02, *p* < .001, η_p_^2^ = .30, reflecting longer RT2 for task-pair switch trials than for task-pair repetition trials (1,018 ms vs. 912 ms; task-pair switch costs: 106 ms). The main effect of task-pair sequence in trial *n* − 1 was significant, too, *F*(1,39) = 4.60, *p* = .038, η_p_^2^ = .11, indicating longer RT2 after task-pair switch trials than after task-pair repetition trials (976 ms vs. 954 ms). Importantly, the interaction of task-pair sequence in trial *n* and task-pair sequence in trial *n* − 1 was also significant, *F*(1,39) = 5.91, *p* = .02, η_p_^2^ = .13. Like for RT1, task-pair switch cost in a current trial were reduced after a task-pair switch compared with after a task-pair repetition (65 ms vs. 146 ms).

A first set of planned simple main effects analyses for this interaction effect demonstrated that task-pair switch costs in the current trial were significant after both a task-pair switch, *F*(1,39) = 10.14, *p* = .003, η_p_^2^ = .21, and a task-pair repetition, *F*(1,39) = 14.71, *p* < .001, η_p_^2^ = .27. Most importantly, a second set of planned simple main effects analyses showed that RT2 in a current task-pair repetition trial was shorter when the previous trial was a task-pair repetition than when it was a task-pair switch (difference: 63 ms), *F*(1,39) = 10.97, *p* = .002, η_p_^2^ = .22. In contrast, RT2 in a current task-pair switch trial was not modulated by the task-pair sequence in trial *n* − 1, *F*(1,39) = 0.80, *p* = .377, η_p_^2^ = .02.

#### Error rates for T2

For the accuracy data, the ANOVA revealed a significant main effect of task-pair sequence in trial *n*, *F*(1,39) = 27.64, *p* < .001, η_p_^2^ = .42, showing that that error rates were higher in task-pair switch trials than in task-pair repetition trials (11.15% vs. 7.18%; task-pair switch costs: 3.9%). The main effect of task-pair sequence in trial *n* −1 was nonsignificant, *F*(1,39) = 2.17, *p* = .149, η_p_^2^ = .05, but the interaction of task-pair sequence in trial *n* and task-pair sequence in trial *n* − 1 was significant, *F*(1,39) = 7.23, *p* = .01, η_p_^2^ = .16, indicating that task-pair switch costs in a current trial were reduced after a task-pair switch compared with a task-pair repetition (2.33% vs. 5.61%).

A first set of planned simple main effects analyses demonstrated that task-pair switch costs in a current trial were significant after both a task-pair switch, *F*(1,39) = 7.15, *p* = .011, η_p_^2^ = .16, and a task-pair repetition, *F*(1,39) = 28.06, *p* < .001, η_p_^2^ = .42. Most importantly and consistent with the RT data, a second set of planned simple main effects analyses showed that error rates in a current task-pair repetition trial were lower when the previous trial was a task-pair repetition than when it was a task-pair switch (difference: 2.60 %), *F*(1,39) = 9.78, *p* = .003, η_p_^2^ = .20. Error rates in a current task-pair switch trial did not differ as a function of the task-pair sequence in trial *n* − 1, *F*(1,39) = 0.51, *p* = .478, η_p_^2^ = .01.

### Discussion

In line with the data reanalysis of previously published task-pair switching studies, we observed task-pair switch costs (e.g., Hirsch et al., 2017, 2021) that were reduced after a task-pair switch relative to after a task-pair repetition. Thus, we confirmed the findings from the data reanalysis in the data of a new experiment. Moreover, additional analyses revealed that performance in a current task-pair repetition was better after a task-pair repetition than after a task-pair switch, but performance in a current task-pair switch did not differ as a function of the task-pair sequence in the previous trial. Hence, there was a task-pair repetition benefit after a task-pair repetition but no task-pair switch benefit after a task-pair switch. As elaborated in the introduction of the present experiment, these findings indicate that repetition priming at the level of task-pair sets, rather than repetition priming at the level of abstract control states, contributes to the sequential adjustment of task-pair switch costs.

## General discussion

The present study aimed to examine whether task-pair set control, as a type of dual-task coordination, is subject to sequential adjustments. To this end, we first reanalyzed the data of four published task-pair switching experiments. In this reanalysis, we found task-pair switch costs and, as the novel finding of this study, a reduction of these costs after a task-pair switch relative to after a task-pair repetition. Second, we confirmed these post hoc findings in a new experiment. In this confirmation experiment, we additionally focused on the exact pattern of the adjustment of the task-pair switch cost and found that performance in a current task-pair repetition was better after a task-pair repetition than after a task-pair switch. In contrast, performance in a current task-pair switch trial did not differ statistically as a function of the task-pair sequence in the previous trial.

### Task-pair switch costs

In line with previous studies, we found task-pair switch costs (e.g., Hirsch et al., 2017, 2021). Thus, the present study provides additional evidence for the notions that the T1 and T2 identities are jointly represented in a single task-pair set and that performing a dual task requires the activation of an appropriate task-pair set in working memory. Going beyond the findings of these published four experiments, Experiment 5 demonstrated that task-pair switch costs also occur in experimental settings that only comprise a long SOA. Thus, the occurrence of task-pair switch costs is not limited to dual-task situations with a strong temporal overlap in T1 and T2 processing, but task-pair sets also seem to play a role in situations with little temporal overlap in T1 and T2 processing (see also Lien & Ruthruff, 2004).

### Sequential adjustment of task-pair switch costs

The novel finding of the present study was the reduced task-pair switch cost after a task-pair switch in comparison to after a task-pair repetition in both the data reanalysis and the new Experiment 5. Thus, like task-order control (Steinhauser et al., 2024; Strobach, 2024a; Strobach et al., 2021a, b, 2023; Strobach & Wendt, 2022), task-pair set control is susceptible to sequential adjustments.

To examine whether repetition priming at the level of abstract control states or/and at the level of task-pair sets contributes to the sequential adjustment of task-pair switch costs, we additionally analyzed in Experiment 5 whether the performance in a current task-pair switch and a current task-pair repetition was modulated by the task-pair sequence in trial *n* − 1. An account assuming repetition priming at the level of abstract control states predicts both enhanced performance in a task-pair switch after a task-pair switch compared with a after task-pair repetition and enhanced performance in a task-pair repetition after a task-pair repetition compared with after a task-pair switch. In contrast, an account assuming repetition priming at the level of task-pair sets predicts that performance in a task-pair repetition trial is better when it follows a task-pair repetition than when it follows a task-pair switch, but it predicts no effect of the previous task-pair sequence on the performance in a current task-pair switch.

The present study showed that the sequential adjustment of task-pair switch costs by the task-pair sequence in the previous trial was mainly driven by a task-pair repetition benefit. That is, performance in a task-pair repetition was better after a task-pair repetition than after a task-pair switch. Importantly, performance in a current task-pair switch did not significantly differ depending on whether the previous trial was a task-pair switch or a task-pair repetition, meaning that a task-pair switch did not enhance a task-pair switch in the following trial. These findings are not consistent with repetition priming effects at the level of abstract control states. Rather, the findings indicate that the sequential adjustment of task-pair switch costs is attributable to repetition priming at the level of task-pair sets.

The absence of a modulation of the performance in a task-pair switch by the task-pair sequence in the previous trial is also inconsistent with an account of general postconflict slowing (e.g., Verguts et al., 2011). General postconflict slowing describes an increase in RTs after participants have experienced a conflict between the task-pairs across two trials. Besides worse performance in a current task-pair repetition after a task-pair switch relative to a task-pair repetition, such a postconflict slowing account predicts that performance in a current task-pair switch is worse after a task-pair switch than after a task-pair repetition. The present study, however, demonstrated a modulating effect of the previous task-pair sequence on the performance in task-pair repetitions but not in task-pair switches.

The notion that repetition priming at the level of task-pair sets contributes to the sequential adjustment of task-pair switch costs is in line with a study by Strobach and colleagues (2021a) who examined sequential adjustments of order switch costs using visual and auditory categorization tasks. Like in the present study, RTs in order-repetition trials were faster when the order-repetition trial followed an order-repetition trial than when it followed an order-switch trial, suggesting repetition priming of order sets as the source of the sequential modulation of order switch costs. In contrast, in a later study with an oculomotor and manual categorization task, Strobach and colleagues (2023) demonstrated enhanced performance in an order-repetition after an order repetition compared with after an order switch and enhanced performance in an order switch after an order switch relative to after an order repetition. Hence, with regard to the sequential adjustment of order control, the existing findings are mixed, and it is unclear which factors have a modulating effect on the impact of the previous order sequence on the performance in a current order switch.

The finding of the present study, that there was no performance benefit in task-pair switches after a task-pair switch compared with after a task-pair repetition, is consistent with findings from task-switching studies. In task-switching studies, participants switch and repeat tasks on a trial-by-trial level, and performance is typically worse in task switches than in task repetitions, an effect referred to as *task-switch costs* (see, e.g., Dreisbach & Heider, 2006; Hirsch et al., 2016; see e.g., Vandierendonck et al., 2018, for a review). Several task-switching studies suggest that preparation for the next trial is task-specific rather than switch-specific (e.g., Koch, 2008; see also Dreisbach et al., 2002). For instance, Koch (2008) used three tasks, and the task sequence (i.e., task switch vs. repetition) was predictable, whereas the task identity was unpredictable in task switches but predictable in task repetitions. Preparation effects occurred in task repetitions, but for task switches with an unpredictable task, there were no preparation effects. Taking into account that other studies observed task-preparation effects in task switches when the identity of the next task was predictable (e.g., Rogers & Monsell, 1995; see Kiesel et al., 2010, for a review), the absence of preparation effects in task-switch trials with an unpredictable task identity indicates that an explicit representation of a switch transition does not improve preparation if the identity of the upcoming task is not known (e.g., Koch, 2008).

Note that there is a crucial difference across the present study and typical studies on sequential adjustments of cognitive control. Whereas typical single-task and dual-task studies focus on conflicts at the level of response selection (e.g., Blais et al., 2014; Schmidt & Houwer, 2011), the present study addressed conflicts at the level of selecting cognitive task representations (i.e., task-pair sets). Thus, the present study focused on a conflict at a different processing level. In addition to studies examining sequential adjustments of task-order control in dual-task contexts (e.g., Strobach et al., 2021a, 2023), we are aware of only one further study that investigated the sequential adjustment of cognitive control after a conflict at the level of selecting cognitive task representations. This study refers to a study by Schuch and Grange (2015).

In this task-switching study, participants switched between three tasks (e.g., A, B, & C). There were *n* − 2 task-repetition sequences in which the task in a given trial *n* was identical to that in trial *n* − 2 (e.g., B in trial *n* − 2, A in trial *n* − 1, and B in trial *n*; i.e., BAB), and *n* − 2 task-switch sequences in which the task in a current trial *n* differed from that in trial *n* − 2 (e.g., CAB). Typically, RTs and error rates in trial *n* are increased in *n* − 2 repetition sequences relative to *n* − 2 switch sequences (e.g., Mayr & Keele, 2000). This effect has been theorized to reflect the persisting inhibition of the task that was abandoned in trial *n* − 1.

Schuch and Grange (2015) found performance to be better after *n* − 2 repetition sequences than after *n* − 2 switch sequences (e.g., the last A task in the end of these sequences BABA vs. CABA). They reasoned that due to the persisting inhibition, trial *n* in *n* − 2 repetition sequences is a trial with a high task-selection conflict. This is because in such trials, the relevant task set is in an inhibited state, and the irrelevant task set is strongly activated due to its use in the previous trial. They proposed that to resolve this interference, cognitive control needs to be upregulated. In the next trial, the persisting upregulation results in a stronger activation of the relevant task set and/or inhibition of the irrelevant task set, facilitating the performance in tasks following a *n* − 2 repetition sequence compared with tasks following a *n* − 2 switch sequence.

Note that in the present study, a task-pair switch after a task-pair switch reflects a *n* − 2 task-pair repetition trial, whereas a task-pair switch after a task-pair repetition reflects a *n* − 2 task-pair switch trial. If a task-pair is inhibited when switching to a new task-pair, performance should be worse in task-pair switches after a task-pair switch than in task-pair switches after a task-pair repetition. However, in the present study, the performance in a task-pair switch did not significantly differ as a function of the task-pair sequence in the previous trial. Thus, the present findings are not consistent with this inhibitory account.

Together with the study by Schuch and Grange (2015) and studies on the sequential adjustment of task-order control in dual-task contexts (e.g., Strobach et al., 2023; Strobach & Wendt, 2022), the findings of the present study suggest that sequential adjustments of cognitive control are not limited to processing conflicts at the level of response selection. Processing conflict at the level of selecting cognitive task representations can also trigger adjustments of cognitive control.

## Summary and conclusions

The findings of the present study show that the control of task-pair sets can be adjusted based on the context. That is, task-pair switch costs are reduced after a task-pair switch than after a task-pair repetition. The data is consistent with the idea that repetition priming effects at the level of task-pair sets account for the observed sequential adjustment of task-pair switch costs, but argue against the involvement of repetition priming effects at the level of abstract control states. Thus, the present study suggests that lower-level memory-based effects contribute to the sequential adjustment of task-pair switch costs. Nevertheless, more research is warranted to examine how top-down cognitive control and priming processes contribute to the sequential adjustment of task-pair switch costs by the task-pair sequence in the previous trial.

## Data Availability

Raw data for all experiments of the data reanalysis and the new Experiment 5 is publicly available in PsychArchives (Experiments 1 & 2: 10.23668/psycharchives.13509, Experiment 3 & 4: http://doi.org/10.23668/psycharchives.3140, Experiment 5: 10.23668/psycharchives.15957). Additional materials will be provided by the corresponding author upon request. None of the experiments was preregistered.

## Appendix 1

The data re-analysis of Experiment 1 to 4 revealed several main effects of experiment and two-way interactions with experiment. Note that these effects are theoretically less interesting for the present study, and that these effects are difficult to interpret due to the violation of the notions of homogeneity of covariance and equality of error variances.

### RT1

In addition to the effects reported above, the ANOVA yielded a significant main effect of experiment, *F*(3, 92) = 10.51, *p* < .001, η_p_^2^ = .26. RT1 was shortest in Experiment 1 (689 ms), followed by Experiment 4 (856 ms), Experiment 2 (1143 ms), and Experiment 3 (1425 ms). A post-hoc analysis revealed that RT1 was significantly shorter in Experiment 1 than in both Experiment 2 (*p* = .003) and Experiment 3 (*p* < .001). Moreover, RT1 was significantly shorter in Experiment 4 than in Experiment 3 (*p* = .01; *p*s for all other comparisons > .074).

Furthermore, there was a significant interaction of experiment and task-pair sequence in trial *n*, *F*(3, 92) = 7.04, *p* < .001, η_p_^2^ = .19. Whereas task-pair switch costs were not significant in Experiment 1 and Experiment 4 (36 ms, *F*(1, 92) = 1.5, *p* = .223, η_p_^2^ = .02, and 18 ms, *F*(1, 92) = 0.38, *p* = .542, η_p_^2^ < .01), there were significant task-pair switch costs in Experiment 2 (106 ms; *F*(1, 92) = 13.01, *p* < .001, η_p_^2^ = .12) and Experiment 3 (187 ms; *F*(1, 92) = 41.6, *p* < .001, η_p_^2^ = .31). Post-hoc tests showed that task-pair switch costs were significantly smaller in Experiment 1 than in Experiment 2 (*p* = .033). In addition, task-pair switch costs in Experiment 4 were significantly smaller than those in Experiment 2 (*p* = .003) and Experiment 3 (*p* = .022; all other *p*s > .051).

There was also a significant interaction of experiment and task-pair sequence in trial *n*-1, *F*(3, 92) = 12.3, *p* < .001, η_p_^2^ = .29. In Experiment 2, RT1 in trial *n* was longer when the previous trial was a task-pair switch than when it was a task-pair repetition (33 ms; *F*(1, 92) = 4.58, *p* = .035, η_p_^2^ = .05). For Experiment 4, there was a reversed data pattern with longer RT1 after task-pair repetitions than after task-pair switches (-84 ms; *F*(1, 92) = 30.79, *p* < .001, η_p_^2^ = .25). In Experiment 1 and Experiment 3, there was no significant effect of the task-pair sequence in trial *n*-1 (Experiment 1: 19 ms; *F*(1, 92) = 1.60, *p* = .209, η_p_^2^ = .02; Experiment 3: 11 ms, *F*(1, 92) = 0.46, *p* = .499, η_p_^2^ = .01). A post-hoc analysis revealed that the data pattern in Experiment 4 significantly differed from that in the other experiments (all *p*s < .001; all other *p*s > .937).

### Error rates in T1

The main effect of experiment was significant, *F*(3, 92) = 6.79, *p* < .001, η_p_^2^ = .65. The error rate was the lowest in Experiment 3 (2.19%), followed by Experiment 2 (3.43%), Experiment 4 (3.69%), and Experiment 1 (5.88%). A post-hoc analysis revealed a significant difference between Experiment 3 and Experiment 1 (*p* = .002; all other *p*s > .08).

Furthermore, the interaction of experiment and task-pair sequence in trial *n *was significant, *F*(3, 92) = 3.97, *p* = .01, η_p_^2^ = .12. In Experiment 1, the error rate was higher in task-pair switch trials than in task-pair repetition trials, resulting in task-pair switch costs of 1.70%, *F*(1, 92) = 10.15, *p* = .002, η_p_^2^ = .09. No significant task-pair switch costs were observed in the other experiments, *F*(1, 92) = 0.52, *p* = .474, η_p_^2^ = .01 for Experiment 2, *F*(1, 92) = 1.14, *p* = .289, η_p_^2^ = .012 for Experiment 3, and *F*(1, 92) = 0.28, *p* = .596, η_p_^2^ < .01. Post-hoc tests showed that there were no significant differences in task-pair switch costs across the experiments (all *p*s > .061). The interaction of experiment and task-pair sequence in trial *n*-1 was not significant, *F*(3, 92) = 1.8, *p* = .153, η_p_^2^ = .06.

### RT2

The ANOVA revealed a significant main effect of experiment, *F*(3, 92) = 13.03, *p* < .001, η_p_^2^ = .30. RT2 was shortest in Experiment 1 (926 ms), followed by Experiment 4 (1049 ms), Experiment 2 (1411 ms), and Experiment 3 (1782 ms). As indicated by a post-hoc analysis, RT2 was significantly shorter in Experiment 1 than in both Experiment 2 (*p* = .008) and Experiment 3 (*p* < .001). In addition, RT2 was shorter in Experiment 4 than in Experiment 3 (*p* < .001; all other *p*s > .082).

The interaction of experiment and task-pair sequence in trial *n* was significant, too, *F*(3, 92) = 12.14, *p* < .001, η_p_^2^ = .28. Whereas there were no significant task-pair switch costs in Experiment 1 (53 ms, *F*(1, 92) = 2.51, *p* = .117, η_p_^2^ = .03) and Experiment 4 (36 ms, *F*(1, 92) = 1.19, *p* = .277, η_p_^2^ = .01), task-pair switch costs were significant in Experiment 2 (169 ms, *F*(1, 92) = 25.4, *p* < .001, η_p_^2^ = .22) and Experiment 3 (288 ms, *F*(1, 92) = 73.77, *p* < .001, η_p_^2^ = .45). A post-hoc analysis revealed that task-pair switch costs were significantly smaller in Experiment 4 than in both Experiment 2 (*p* = .002) and Experiment 3 (*p* < .001). Moreover, task-pair switch costs in Experiment 1 were smaller than those in Experiment 2 (*p* = .013) and Experiment 3 (*p* = .002; all other *p*s > .347).

In addition, the interaction of experiment and task-pair sequence in trial *n*-1 was significant, *F*(3, 92) = 16.17, *p* < .001, η_p_^2^ = .35. In Experiment 1 and Experiment 3, there was no significant effect of the task-pair sequence in trial *n*-1 (Experiment 1: 20 ms, *F*(1, 92) = 1.22, *p* = .273, η_p_^2^ = .01; Experiment 3: 27 ms, *F*(1, 92) = 2.28, *p* = .134, η_p_^2^ = .02). In Experiment 2, RT2 was longer after a task-pair switch than after a task-pair repetition in the previous trial (63 ms, *F*(1, 92) = 11.74, *p* < .001, η_p_^2^ = .11). Experiment 4 showed a reversed data pattern with longer RTs after task-pair repetitions than after task-pair switches (-106 ms, *F*(1, 92) = 33.29, *p* < .001, η_p_^2^ = .27). A post-hoc analysis revealed that the data pattern in Experiment 4 significantly differed from that in all other experiments (all *p*s < .001; all other *p*s > .587).

### Error rates in T2

The main effect of experiment, *F*(3, 92) = 2.36, *p* = .072, η_p_^2^ = .07, the interaction of experiment and task-pair sequence in trial *n*-1, *F*(3, 92) = 0.95, *p* = .42, η_p_^2^ = .03, and the interaction of experiment and task-pair sequence in trial *n*, *F*(3, 92) = 2.28, *p* = .084, η_p_^2^ = .02, were not significant.

## Footnotes

1 So far, task-pair set control and component-task order control has primarily been examined in independent research lines. Thus, it is currently an open question of whether component-task identity and component-task order information is represented in a single representation or two separate representation (see Hirsch & Koch, 2024, for a discussion on different organizations).

2 A post hoc analysis revealed that in Experiment 2, the sequential adjustment of task-pair switch costs was only significant for RT2, *F*(1,92) = 4.57, *p* = .035, η_p_^2^ = .05; RT1, *F*(1,92) = 0.16, *p* = .689, η_p_^2^ < .01; error rates in T1, *F*(1,92) = 0.43, *p* = .516, η_p_^2^ = .01; error rates in T2, *F*(1,92) = 0.28, *p* = .596, η_p_^2^ < .01. In contrast, the sequential adjustment of task-pair switch costs was significant for RT1 and RT2 in Experiment 1, *F*(1,92) = 9.6, *p* = .003, η_p_^2^ = .09, and *F*(1,92) = 4.23, *p* = .043, η_p_^2^ = .04; error rates in T1, *F*(1,92) = 2.1, *p* = .15, η_p_^2^ = .02; error rates in T2, *F*(1,92) = 2.11, *p* = .15, η_p_^2^ = .02; for RT1, RT2, and error rates of T2 in Experiment 3, *F*(1,92) = 4.73, *p* = .032, η_p_^2^ = .05, *F*(1,92) = 7.96, *p* = .006, η_p_^2^ = .08, and *F*(1,92) = 4.33, *p* = .04, η_p_^2^ = .05; error rates in T1, *F*(1,92) = 1.77, *p* = .186, η_p_^2^ = .02, and for RT1, RT2, and error rates of T2 in Experiment 4, *F*(1,92) = 14.22, *p* < .001, η_p_^2^ = .13, *F*(1,92) = 19.51, *p* < .001, η_p_^2^ = .18, and, *F*(1,92) = 8.76, *p* = .004, η_p_^2^ = .09; error rates in T1, *F*(1,92) = 1.15, *p* = .287, η_p_^2^ = .01.

3 Note that we did not calculate the second set of simple main effect analyses because the main aim of the data reanalysis was to establish this novel effect at all. Instead, we calculated this second set in Experiment 5 in which we elaborated on the mechanisms underlying the sequential adjustment of task-pair control in a new data set.

## Code availability

Codes will be provided by the corresponding author upon request.
